# A Rare Co-occurrence of Lumbo-Costo-Vertebral Syndrome With Congenital Lumbar Hernia in a Six-Year-Old Child

**DOI:** 10.7759/cureus.65308

**Published:** 2024-07-24

**Authors:** Rishabh Dhabalia, Shivali V Kashikar, Pratapsingh Parihar, Komal Mishra, Riya Yadav, Shivani S Bothara

**Affiliations:** 1 Radiodiagnosis, Datta Meghe Institute of Higher Education & Research, Wardha, IND

**Keywords:** congenital, hemivertebrae, lcvs, lumbar hernia, lumbo-costo-vertebral

## Abstract

Lumbo-costo-vertebral syndrome (LCVS) is a very rare congenital disorder seen in children. It is characterized by a congenital absence of ribs, vertebral anomalies, scoliosis, meningocele, and hypoplastic abdominal wall muscles presenting as abdominal wall hernia. We present a case of a six-year-old Indian female who came with complaints of swelling in the left lumbar region since birth, which was evident in coughing and scoliosis. On auscultation, bowel sounds were heard over the swelling. Physical examination revealed a left lumbar hernia and scoliosis. Abdominal X-rays revealed the absence of the 12th rib on the left side and vertebral anomalies with kyphoscoliosis. Abdominal ultrasonography (USG) showed a left lumbar hernia with bowel loops as its content. Computed tomography (CT) was done, which confirmed the X-ray and USG findings. Based on clinical and radiological findings, a diagnosis of LCVS associated with congenital lumbar hernia (CLH) was made. The patient was then referred to the surgery department for further management. This case illustrates a unique link between two extremely rare conditions and emphasizes the necessity of thorough clinical and radiological evaluation in suspected patients for early diagnosis and treatment.

## Introduction

Lumbo-costo-vertebral syndrome (LCVS) is a rare congenital condition seen primarily in the pediatric age group. It is characterized by lumbar hernia, agenesis of ribs, and vertebral anomalies like hemivertebrae and scoliosis [1]. Vascular disruption of somites is believed to be the cause of various associated defects [2]. Congenital lumbar hernia (CLH) is a seldom documented entity [3] that can occur in the superior lumbar triangle (Grynfeltt-Lesshaft triangle), the inferior lumbar triangle (Petit triangle), diffusely or outside of those triangles in the lumbar region [4]. CLH can be associated with other congenital anomalies involving vertebrae, ribs, kidneys, and muscles, making it a rare condition [5]. It may be present at birth or identified later in life [6]. Patients usually present with lower backache. Small lumbar hernias may be asymptomatic and become evident upon crying [7]. LCVS can also be associated with VACTERL anomalies, which are extremely rare [8]. Suspected patients should be thoroughly evaluated by clinical examination, X-ray, USG, echocardiography, CT, and MRI to rule out other associated anomalies. Early diagnosis and management are necessary to prevent complications like incarceration and strangulation of herniated bowel loops in the case of CLH [9]. Thus, early surgical repair of CLH defect is recommended [4]. We present a very unique case of a six-year-old girl with an exceptionally rare association of two uncommon entities, LCVS and CLH.

## Case presentation

**Figure 1:**
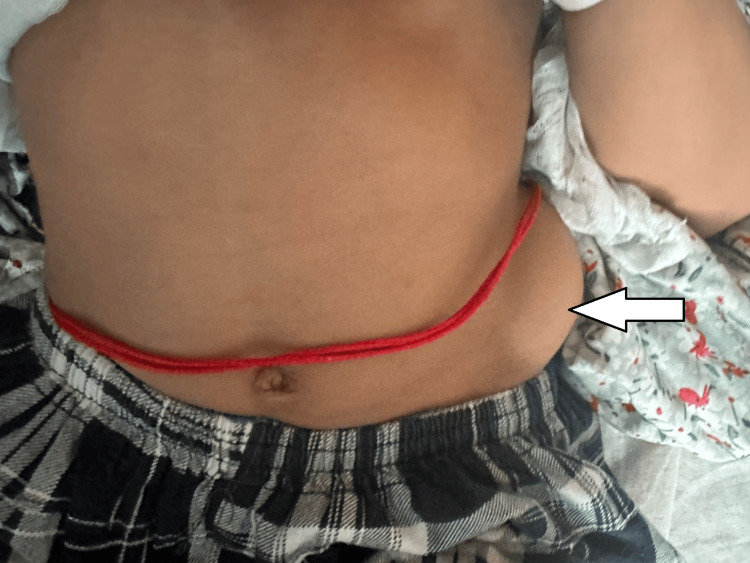
Swelling in the left lumbar region (white arrow)

**Figure 2:**
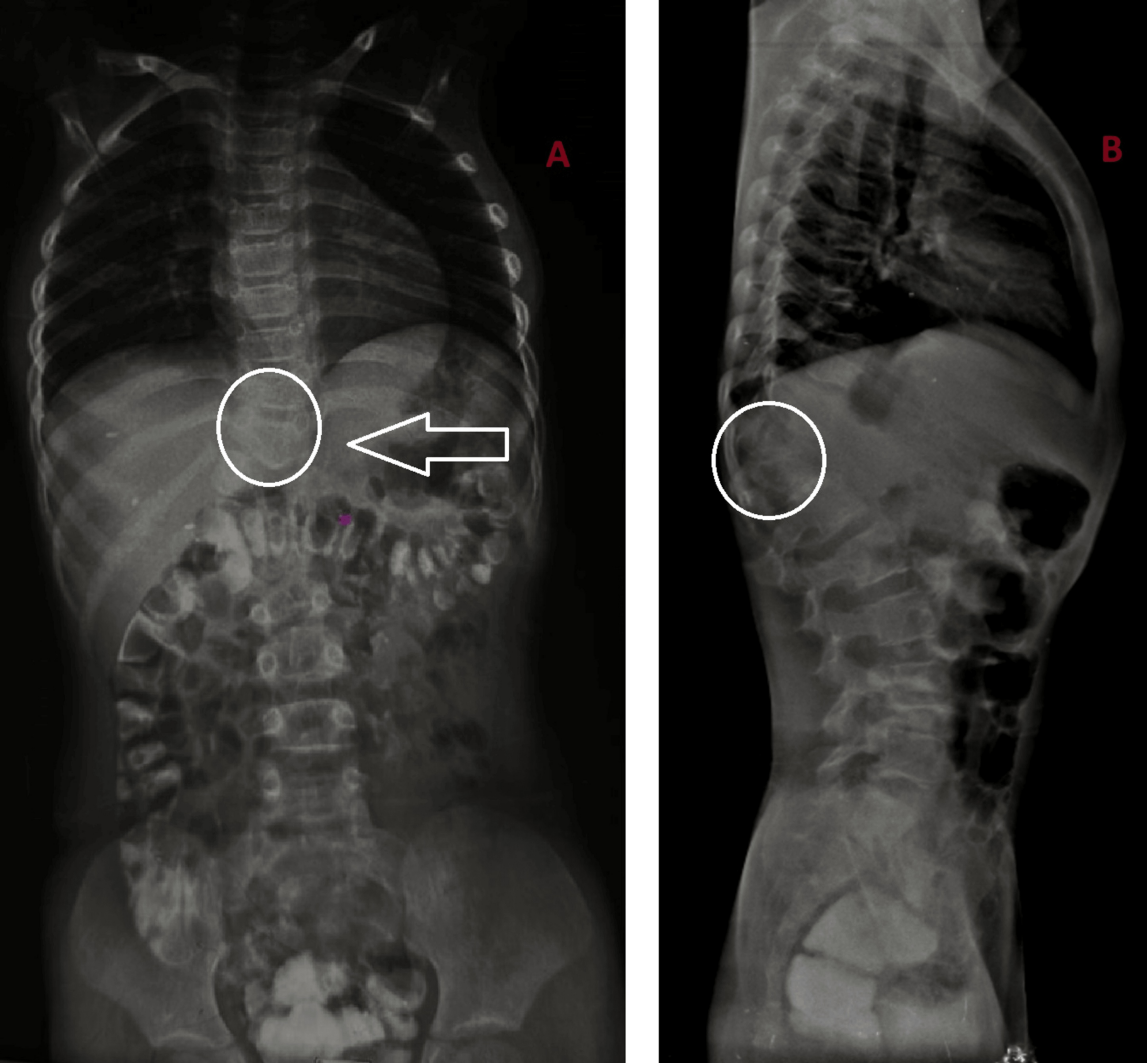
Frontal (A) and lateral (B) X-ray abdomen showing dorsal vertebral anomalies and dextroscoliosis (circle) with absence of left 12th rib (arrow)

**Figure 3:**
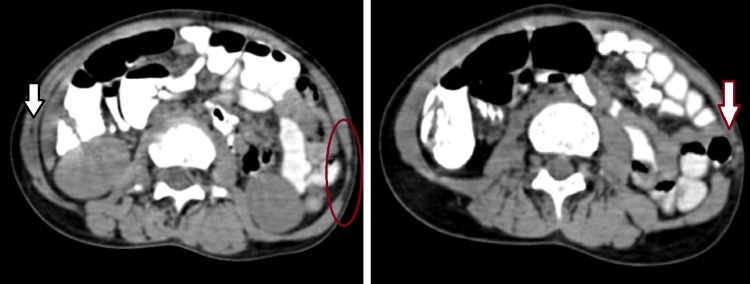
CT abdomen axial sections showing hypoplastic, thinned-out abdominal wall muscles (red circle) in the left lumbar region with herniation of bowel loops through the defect (red arrow) as compared to the normal musculature on the right side (black arrow) CT - computed tomography

**Figure 4:**
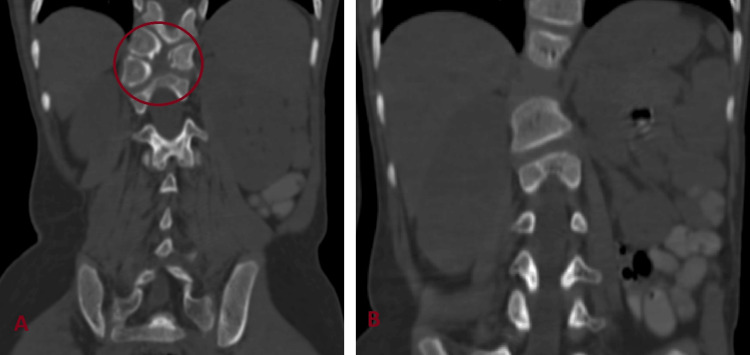
CT coronal sections in bone window showing (A) butterfly vertebra and hemivertebra at D11 and D12 level respectively (red circle) and (B) dextroscoliosis CT - computed tomography

**Figure 5:**
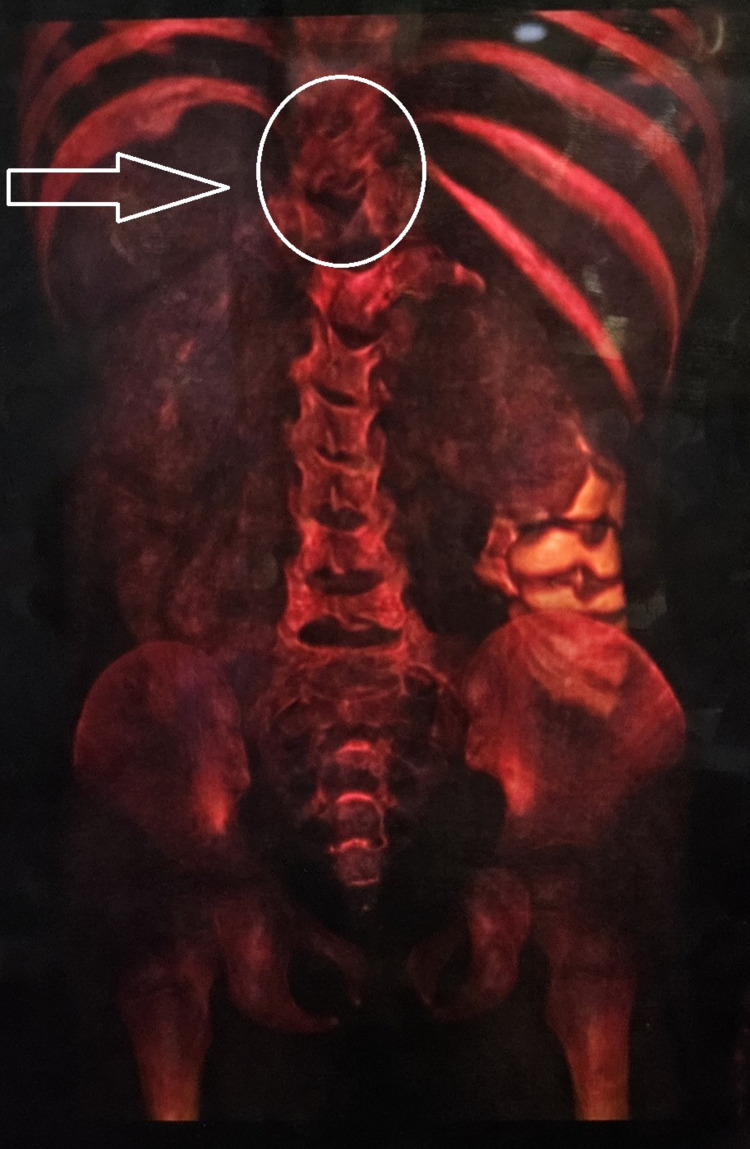
3D-volume rendered CT image showing vertebral anomalies (circle) with an absence of the 12th rib on the left side (arrow) CT - computed tomography

A six-year-old girl was brought by her parents with complaints of left lumbar swelling since birth. Initially, it was small, but gradually, it kept increasing in size to attain the present size of approximately 6 x 4 cm (Figure 1). The swelling was prominent when the patient was laughing, coughing, or crying and decreased in size in the supine position. On physical examination, a painless, rounded, soft, and reducible swelling was noted in the left lumbar region. On auscultation, bowel sounds were heard over the swelling. Frontal and lateral X-ray-abdomen showed dorsal vertebral anomalies with dextroscoliosis and absent 12th rib on the left side (Figure 2). USG abdomen revealed an abdominal wall defect of approximate size 1.5 cm in the left lumbar region with herniation of bowel loops suggestive of lumbar hernia. CT of the abdomen and pelvis showed hypoplastic, thinned-out abdominal wall muscles with herniation of bowel loops through a defect of approximate size 1.5 cm in the left lumbar region (Figure 3). Vertebral anomalies were noted in the form of butterfly vertebra and hemivertebra at D11 and D12 vertebral level, respectively, with dextroscoliosis and absent left 12th rib (Figure 4, 5). Based on the clinical and radiological findings, a diagnosis of LCVS associated with CLH was made. Our patient was then referred to the surgery department for lumbar hernia repair.

## Discussion

LCVS is a rare disorder primarily seen in children [1]. The anomalies are believed to develop due to an abnormality during somatic differentiation during embryogenesis between the third and fifth week of gestational age [10]. It is characterized by abnormalities of the vertebrae, agenesis of ribs, and hypoplasia of abdominal wall musculature, which may present as congenital lumbar hernias (CLH) [1]. Other anomalies like congenital heart disease, inguinal hernia, renal agenesis, and congenital talipes equinovarus (CTEV) might also be associated with it [11]. Abnormalities or absence of 11th and 12th ribs can lead to weakened attachment of adjacent abdominal wall muscles, predisposing the patient to the risk of developing congenital lumbar hernias. LCVS is the most common congenital anomaly associated with CLH, yet the combination is rarely reported. It may also be associated with VACTERL anomalies, as Harris et al. (2009) reported in a term neonate [8].

LCVS can be present at birth or identified later in life [6]. CLH may remain asymptomatic initially and gradually increase in size enough to become evident when the child is coughing, laughing, or crying [7]. Vertebral anomalies, agenesis of ribs, and scoliosis can be detected on X-ray. On the USG-abdomen, lumbar hernia and its content can be detected. CT or magnetic resonance imaging (MRI) of the abdomen-pelvis is the investigation of choice done to confirm the X-ray and USG findings, to know hernial sac contents and muscular hypoplasia, and to rule out additional anomalies [12]. The hernial sac's contents usually include the small or large bowel, mesentery, and omental fat and rarely can the ovary, spleen, or kidney be found within the hernial sac [7]. In our patient, X-rays revealed anomalies of ribs and vertebrae with scoliosis. USG showed a left-sided lumbar hernia with bowel loops as content. CT revealed hypoplastic and thinned-out abdominal wall muscles in the left lumbar region with bowel loops herniating through a defect of approximate size 1.5 cm, dorsal hemivertebra, and butterfly vertebra with kyphoscoliosis and absence of left 12th rib.

Surgery is recommended for the management of CLH, although laparoscopy plays a role nowadays [13]. Elective surgical repair before 12 months of age is recommended as the defect size may grow in size and complicate primary direct closure [14]. Early repair can also prevent complications like incarceration and strangulation of herniated bowel loops [15, 16].

## Conclusions

We have discussed an extremely rare case of LCVS associated with CLH in a six-year-old girl who presented with left lumbar swelling since birth. Her X-ray of the abdomen showed vertebral anomalies with dextroscoliosis and an absent 12th rib on the left side. USG abdomen revealed a left-sided lumbar hernia with bowel loops as its content. CT abdomen-pelvis confirmed the X-ray and USG findings.

LCVS and CLH are two extremely rare conditions that can sometimes be linked and manifest together. No specific gene defect has been detected; hence, it remains solely a clinical diagnosis. Consequently, thorough clinical and radiological evaluation is crucial for early diagnosis and treatment. Early surgical repair is recommended for uneventful primary closure of the hernia defect and prevention of complications like incarceration and strangulation of herniated bowel loops.
